# Effect of Semi-Conductive Layer Modified by Magnetic Particle SrFe_12_O_19_ on Charge Injection Characteristics of HVDC Cable

**DOI:** 10.3390/polym11081309

**Published:** 2019-08-05

**Authors:** Yanhui Wei, Mingyue Liu, Jiaxing Wang, Guochang Li, Chuncheng Hao, Qingquan Lei

**Affiliations:** Institute of Advanced Electrical Materials, Qingdao University of Science and Technology, Qingdao 266042, China

**Keywords:** HVDC cable, semi-conductive composites, SrFe_12_O_19_, space charge accumulation

## Abstract

For high voltage direct current (HVDC) cable, a semi-conductive layer lies between the conductor and the insulation layer; as the charge migrates the path from the conductor to the insulation material, it will affect space charge injection. In this work, the research idea of changing the injection path of moving charges within semi-conductive layer by magnetic particles was proposed. Semi-conductive composites with different SrFe_12_O_19_ contents of 1 wt.%, 5 wt.%, 10 wt.%, 20 wt.%, and 30 wt.% were prepared, and the amount of injected charges in the insulation sample was characterized by space charge distribution, polarization current, and thermally-stimulated depolarization current. The experimental results show that a small amount of SrFe_12_O_19_ can significantly reduce charge injection in the insulation sample, owing to the deflection of the charge migration path, and only part of the electrons can enter the insulation sample. When the content is 5 wt.%, the insulation sample has the smallest charge amount, 0.89 × 10^−7^ C, decreasing by 37%, and the steady-state current is 6.01 × 10^−10^ A, decreasing by 22%. When SrFe_12_O_19_ content exceeds 10 wt.%, the charge suppression effect is not obvious and even leads to the increase of charge amount in the insulation sample, owing to the secondary injection of charges. Most moving charges will deflect towards the horizontal direction and cannot direct access to the insulation sample, resulting in a large number of charges accumulation in the semi-conductive layer. These charges will seriously enhance the interface electric field near the insulation sample, leading to the secondary injection of charges, which are easier to inject into the insulation sample.

## 1. Introduction

Space charge accumulation and suppression methods of high voltage direct current (HVDC) cable have been hot research topics with the development and application of higher voltage grade cable [1]. Space charge accumulation in an insulation layer will cause local electric field distortion, accelerating the degradation and aging of the insulation material [2,3,4,5]. A semi-conductive shielding layer as an essential component of HVDC cable plays an important role in uniform electric field and makes the conductor wire core and the insulation layer connect tightly [6,7,8,9,10,11,12]. Besides, it is also a direct path of charge injected from the conductor to the insulation layer, which has a great impact on charge injection and accumulation in the insulation layer [10,11,13].

As for the space charge problem of HVDC cables, there have many works focusing on the insulation materials low-density polyethylene (LDPE) and cross-linked polyethylene (XLPE) in past two decades, which mainly include the charge transport characteristics and the modification of insulating materials [14,15,16,17,18,19,20,21,22,23]. The most representative working was the study of nanocomposites, where the space charge can be effectively suppressed when a small amount of inorganic nano-particles are doped into the polymer matrix, while nanocomposite materials are still far from the application of higher voltage grade cable due to the limitation of the purity of insulation material. 

In recent years, there also have some studies that pay attention to the semi-conductive layer of HVDC cable. A semi-conductive layer is a kind of composite, which is mainly composed of carbon black (CB), ethylene-vinyl acetate copolymer (EVA), polyethylene (PE). At present, the related researches mainly focused on the modification of the semi-conductive material by adjusting the filler ratio, type, or adding the second filler [24,25,26,27,28,29,30,31]. The objective was to improve surface finish or inhibit a positive temperature coefficient (PTC) effect, while the charge suppression effect was not obvious. If one can control the path of moving charges in the semi-conductive layer, the charges injected from the metal to the insulation layer can be effectively suppressed. 

**Figure 1:**
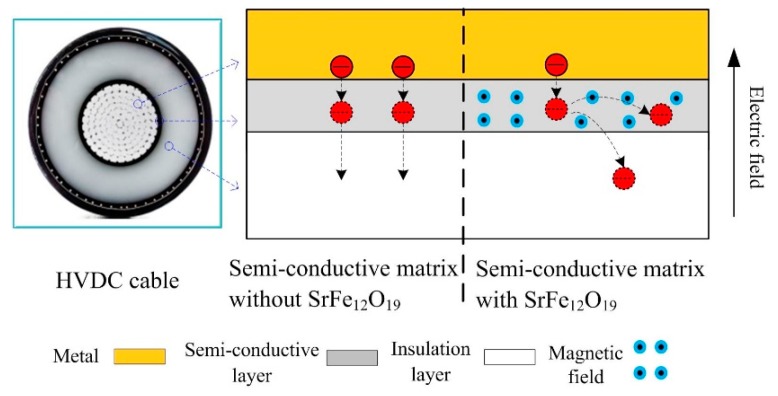
Schematic diagram of research idea. HDVC = high voltage direct current.

SrFe_12_O_19_, as an important magnetic particle, has been widely used in information recording, electronic communication and electronic equipment [32]. Some composite materials based on a polymer matrix, such as SrFe_12_O_19_/PA6, SrFe_12_O_19_/PP, and SrFe_12_O_19_/PVC, have been reported in study of the magnetic properties of composite materials [33,34,35,36,37]. However, the effect of Lorentz force generated by magnetic particles on moving charges in composites has never been studied. According to the electromagnetic field theory, the moving charges in the magnetic field are affected by Lorentz force, and their moving path will be changed. Therefore, the research idea of changing the injection path of moving charges within semi-conductive layer by magnetic particles was proposed, as shown in Figure 1.

In this paper, semi-conductive composites with different SrFe_12_O_19_ contents of 1 wt.%, 5 wt.%, 10 wt.%, 20 wt.%, and 30 wt.% were prepared. SrFe_12_O_19_/semi-conductive composites characterizations, including infrared spectrum analysis, the SEM and element analysis, hysteresis loops, and resistivity, are introduced in Section 2. A simplified cable structure ‘Metal electrode—Semi-conductive composite—Insulation sample—Metal electrode’ (M-S-I-M) was used to measure space charge distribution, polarization current, and thermally-stimulated depolarization current in the insulation sample, as shown in Section 3. The charge injection characteristics in the insulation sample under the action of semi-conductive layer with different SrFe_12_O_19_ contents was compared, and the mechanism was analyzed, which is shown in Section 4.

## 2. Sample Preparation and Characterization

### 2.1. Sample Preparation

**Figure 2:**
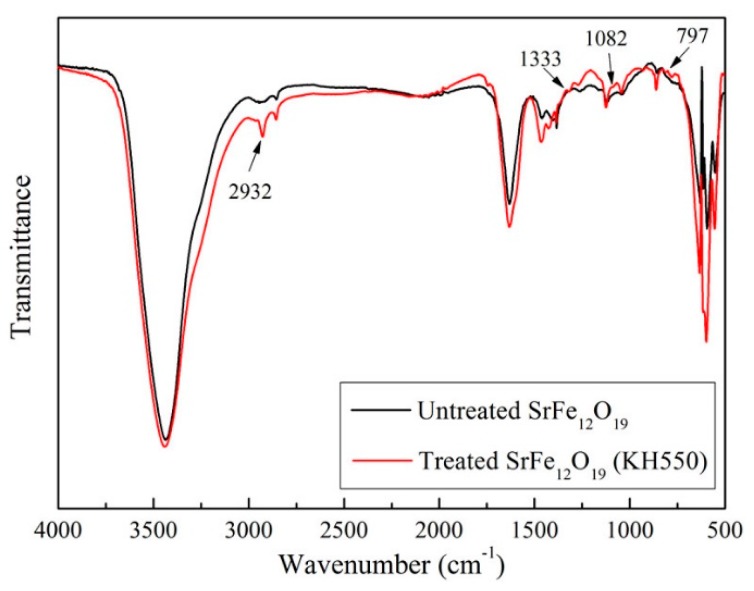
Infrared spectrum analysis of SrFe_12_O_19_ particles before and after modification with KH550.

The semi-conductive matrix (Borealis, Europe) used in the experiment is a semi-conductive composite for HVDC cable, which is composed of CB, EVA, PE. SrFe_12_O_19_ with a size of 800 nm produced by Aladdin Industrial Corporation (Shanghai, China) was used. In order to avoid agglomeration, improve the dispersion of SrFe_12_O_19_ particles in the semi-conductive matrix and lower the interface between the two-phase materials, surface modifications of SrFe_12_O_19_ particles were firstly implemented using silane coupling agent KH550 before mixing. The interaction between the surface of SrFe_12_O_19_ particles and the coupling agent was analyzed by infrared spectroscopy, as shown in Figure 2.

As can be seen from Figure 2, an absorption peak appears near 2392 cm^−1^, which is the characteristic absorption peak in stretch vibration of CH_2_ in the coupling agent. The C-N peak appears at 1333 cm^−1^, and the characteristic peak of the Si-O-Si long chain appears at 1082 cm^−1^. In addition, there are many peaks below 1000 cm^−1^, which are out-of-plane bending vibration peaks of C-H. The results show that the surface of SrFe_12_O_19_ particles were successfully modified by the KH550.

SrFe_12_O_19_/semi-conductive composites were prepared by the melt blending method. Prior to the preparation, the modified SrFe_12_O_19_ and semi-conductive composite were dried in a vacuum chamber. Firstly, the semi-conductive matrix was blended in the internal mixer at 135 °C for 5 min, and the desired amount of SrFe_12_O_19_ was added to the internal mixer and blended for 15 min. Five kinds of composites with different SrFe_12_O_19_ contents were obtained. Secondly, the films of SrFe_12_O_19_/semi-conductive composites were prepared by the vulcanizing press. After that, the samples was cooled for 10 min at 10 MPa. Finally, SrFe_12_O_19_/semi-conductive composites with five different contents of 1 wt.%, 5 wt.%, 10 wt.%, 20 wt.%, and 30 wt.% were obtained. Adopting the same method, the insulation sample, XLPE, with a thickness of 300 μm was prepared.

### 2.2. Characterization of SrFe_12_O_19_/Semi-Conductive Composites

#### 2.2.1. Micromorphology 

**Figure 3:**
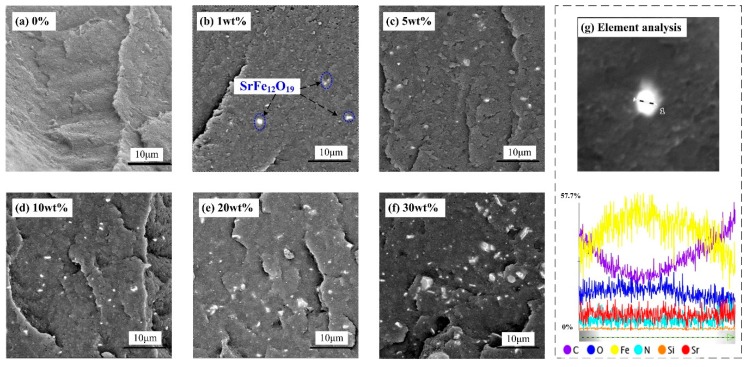
The SEM images and element analysis of SrFe_12_O_19_/semi-conductive composites. (**a**) 0%; (**b**) 1 wt.%; (**c**) 5 wt.%; (**d**) 10 wt.%; (**e**) 20 wt.%; (**f**) 30 wt.%; (**g**) element analysis.

The cross-section micromorphology of composites were observed. Figure 3 shows the SEM images and element analysis of SrFe_12_O_19_/semi-conductive composites.

Figure 3a–f show SEM images of cross sections of SrFe_12_O_19_/semi-conductive composites with the contents of 0%, 1 wt.%, 5 wt.%, 10 wt.%, 20 wt.%, and 30 wt.%, respectively. It can be observed that the SrFe_12_O_19_ particles are well-dispersed in the semi-conductive matrix, as shown in the blue region marker in Figure 3b. The highlighted particles in Figure 3a–f are analyzed by energy spectrum analysis. As shown in Figure 3g, the content of Fe element across the test line is the highest, about 57.7%, followed by the C and O element, and the content of C is the highest at both ends of the test line. The test results are consistent with the SEM observation of the test area in Figure 3g. It can be confirmed that SrFe_12_O_19_ particles were well blended with the semi-conductive matrix. 

#### 2.2.2. Hysteresis Loop of SrFe_12_O_19_/Semi-Conductive Composites

**Figure 4:**
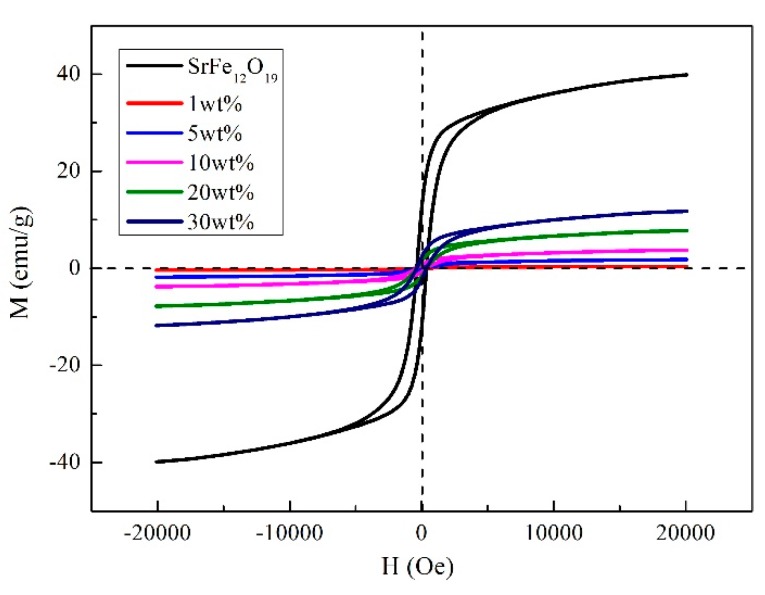
Hysteresis loops of SrFe_12_O_19_/semi-conductive composites.

The hysteresis loops of SrFe_12_O_19_ particles and SrFe_12_O_19_/semi-conductive composites with different doping contents at room temperature were measured, as shown in Figure 4.

The measured results show that the residual magnetization of pure SrFe_12_O_19_ particles is 12.24 emu/g. The residual magnetization of SrFe_12_O_19_/semi-conductive composites are 0.078 emu/g (1 wt.%), 0.36 emu/g (5 wt.%), 0.73 emu/g (10 wt.%), 1.52 emu/g (20 wt.%), and 2.31 emu/g (30 wt.%). 

Compared with pure SrFe_12_O_19_ particles, the residual magnetization intensity of SrFe_12_O_19_/semi-conductive composite decreases. At the same time, it can be found that the more doped SrFe_12_O_19_ particles are, the greater the residual magnetization intensity. Because magnetic particles are surrounded by the semi-conductive matrix, its surface energy is reduced, resulting in difficulty of the magnetization orientation process. Besides, the CB particles in semi-conductive matrix have some diamagnetism. Finally, the doping concentration of SrFe_12_O_19_ particles is proportional to the magnetization intensity.

#### 2.2.3. Resistivity of SrFe_12_O_19_/Semi-Conductive Composites

**Figure 5:**
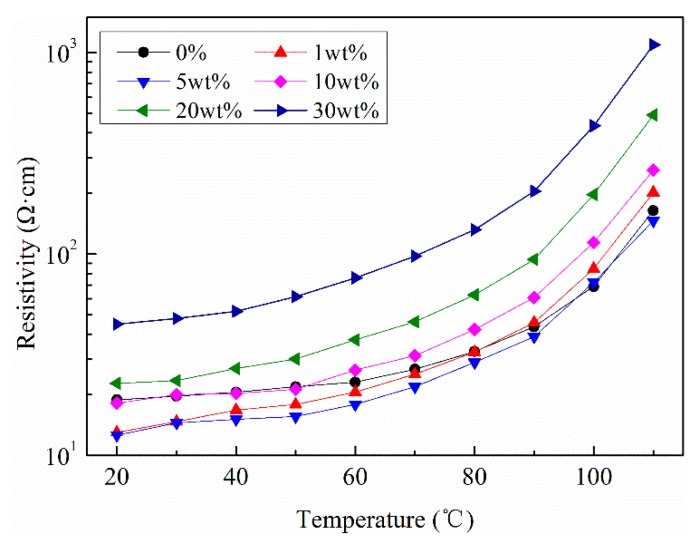
Resistivity of SrFe_12_O_19_/semi-conductive composites versus temperature.

Resistivity of SrFe_12_O_19_/semi-conductive composites were measured at different temperatures by semi-conductvie resistance test device, as shown in Figure 5. 

It can be seen that the resistivity of composites with contents of 0%, 1 wt.%, and 5 wt.% do not change obviously, showing a slightly decrease in partial enlarged drawing, while when SrFe_12_O_19_ particles exceeds 10 wt.%, the resistivity increases significantly. That is mainly due to the fact that the resistivity of SrFe_12_O_19_ particles is much higher than that of CB particles. In semi-conductive composite without SrFe_12_O_19_ particles, its resistivity mainly depends on CB particles. When SrFe_12_O_19_ particles are doped, the original conductive channel will be blocked and the resistivity will increase. Therefore, the higher the doping contents of SrFe_12_O_19_ particles, the greater the resistivity of composites. In addition, it can be found that the PTC effect becomes evident when the temperature exceeds 90 °C, while for low doping concentration of 1 wt.% and 5 wt.%, the change of resistivity slowly rises with the increasing temperature. This indicates that a small amount of magnetic particles doping cannot affect the PTC effect of the semi-conductive matrix.

## 3. Experimental Results and Analysis

### 3.1. Space Charge Characteristics

Space charges in insulation material stressed by DC voltage mainly comes from two parts: homo-charges generated by the injection of electrode and the hetero-charges generated by the ionization of impurity particles [38]. For PE, when the electric field exceeds about 10 kV/mm, the injected charges from the electrodes are dominated, which are the main source of the accumulated charges in the insulation material. 

**Figure 6:**
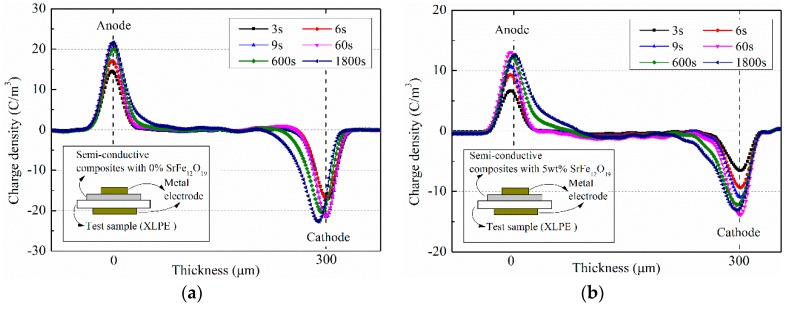
Space charge distribution in insulation sample under the action of semi-conductive composites with different SrFe_12_O_19_ contents (**a**) 0%; (**b**) 5 wt.%.

**Figure 7:**
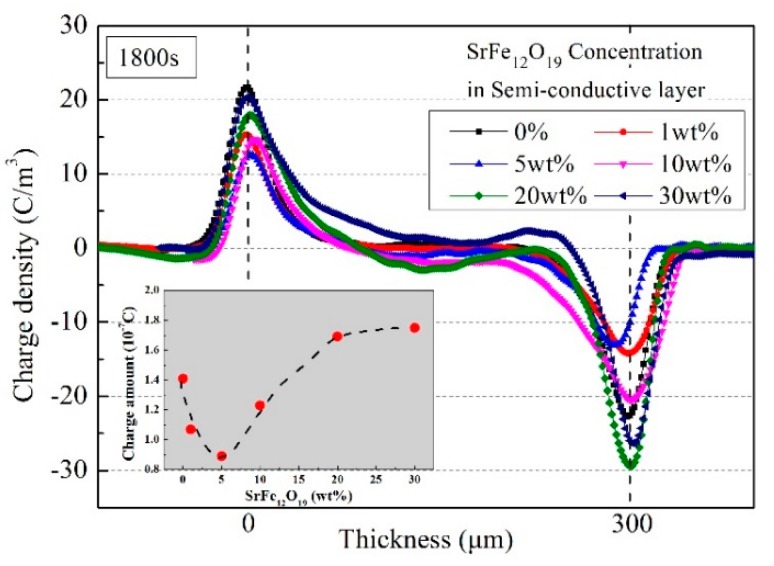
Comparison of space charge distributions at 1800 s in in insulation sample under semi-conductive composites with different SrFe_12_O_19_ contents.

The pulse electro-acoustic (PEA) method can be used to intuitively observe space charge distribution in the insulation material. For the method, the semi-conductive layer between the metal electrode and the insulation material plays the role of acoustic impedance matching, meanwhile it will affect the charge injection in the insulation material. In the experiments, semi-conductive composite materials with different SrFe_12_O_19_ contents were used as the semi-conductive layer in the PEA test, and XLPE films with the same conditions were used as the test samples. The applied electric field was 20 kV/mm, keeping time for 1800 s. The influence of SrFe_12_O_19_/semi-conductive composites on the space charge distribution of the same insulation layer were observed. Figure 6 shows space charge distribution in XLPE under semi-conductive composites with SrFe_12_O_19_ contents of 0% and 5 wt.%. Figure 7 shows space charge distributions at 1800 s in XLPE under semi-conductive composites with different SrFe_12_O_19_ concentrations.

As can be seen from Figure 6, the charges near interface between the electrode and the insulation sample are reduced under the action of the semi-conductive layer doped with a small amount of SrFe_12_O_19_. Generally, these charges near the electrode are mainly composed of the induced charge and the injected charge. Keeping the same applied electric field, the induced charges near the electrode will remain unchanged. Hence, the difference of the charges near the electrode mainly come from the injected charge from the electrode. Comparing Figure 6a,b, for the semi-conductive composites without SrFe_12_O_19_, the maximum charges near the two electrodes are 21.75 C/m^3^ and 22.77 C/m^3^, respectively. After doping by SrFe_12_O_19_ with 5 wt.%, the interface charges decrease to 13.11 C/m^3^ and 14.03 C/m^3^, respectively. This is because when the charges injected from the metal electrode to the insulating sample pass through the SrFe_12_O_19_/semi-conductive composites, the moving charges will be affected by the horizontal Lorentz force, leading to the deflection of the charge migration path. In addition, the moving charges are subjected to the electric field force in the vertical direction, and only some of the electrons enter the insulation sample.

Space charge distributions in the insulation sample at 1800 s were compared under the action of semi-conductive layer with different SrFe_12_O_19_ contents, as shown in Figure 7. It can be seen that the accumulated charges in the insulation samples firstly decreases and then increases with the increase of SrFe_12_O_19_ content. When the content is 5 wt.%, the insulation sample has the minimum charges, 0.89 × 10^−7^ C, reduced by about 37% than that without doping. When SrFe_12_O_19_ content is high, the charge amount in the insulation sample increases. It reaches 1.75 × 10^−7^ C at 30 wt.%, increased by about 24% than that without doping.

When SrFe_12_O_19_ content is high, a large Lorentz force will be generated in the SrFe_12_O_19_/semi-conductive composites, most moving charges will deflect towards the horizontal direction, and cannot direct access to the insulation sample, resulting in a large number of charges accumulation in the semi-conductive layer. These accumulated charges will cause local electric field distortion, which will enhance the interface electric field between the semi-conductive layer and the insulation sample, leading to the secondary injection of charges, which are easier to inject into the insulation sample.

### 3.2. Polarization and Depolarization Current Characteristics

**Figure 8:**
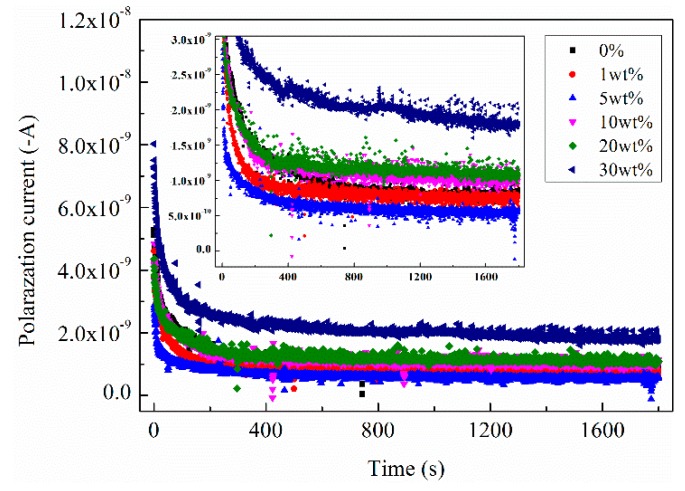
Polarization current in insulation sample under semi-conductive composites with different SrFe_12_O_19_ contents.

The polarization current can reflect the charge transport characteristics of the insulation sample during applying voltage [5,39]. The depolarization current can reflect the trapped charges in the insulation sample, which mainly come from the charge’s injection under the strong electric filed [5,39]. In the experiment, the semi-conductive layer modified with different SrFe_12_O_19_ was placed between the metal electrode and the insulating sample, forming the structure of ‘M-S-I-M’. The polarization current passing through the insulation sample under the action of the modified semi-conductive layer was measured by electrometer. After removing the voltage, the thermal stimulation depolarization current was measured by the Novocontrol system. Figure 8 shows polarization current of XLPE under semi-conductive composites with different SrFe_12_O_19_ concentrations.

It can be seen from Figure 8, when SrFe_12_O_19_ content is low, the current in the insulation sample decreases to some extent. It is 6.01 × 10^−10^ A for the content of 5 wt.%, which is about 22% lower than that without doping. When SrFe_12_O_19_ content exceeds 10 wt.%, the current gradually increases with the increase of the magnetic particle content. Especially when the content is high, the current increases significantly, reaching 1.80 × 10^−9^ A for 30 wt.%. 

**Figure 9:**
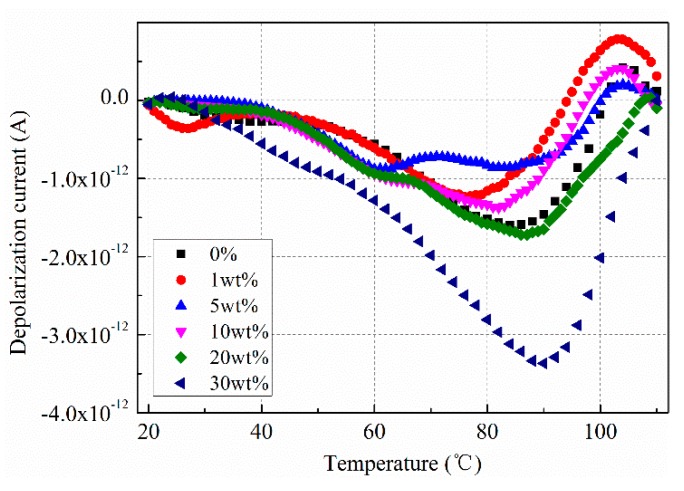
Depolarization current in insulation sample under semi-conductive composites with different SrFe_12_O_19_ contents.

In order to further verify the influence of SrFe_12_O_19_ content on the charge injection of insulation sample, after removing the voltage, the thermal stimulation depolarization current of the insulation sample was measured. In the test, the heating rate was set to 1 °C/min, and the heating range changes from 20 °C to 100 °C. The test results are shown in Figure 9.

It can be seen that the depolarization current in the insulation sample is significantly different for different SrFe_12_O_19_ content. When SrFe_12_O_19_ content is low, the depolarization current is less than that without doping. The maximum current is 8.52 × 10^−13^ A for 5 wt.%. When SrFe_12_O_19_ content exceeds 20 wt.%, the current significantly increases, it reaches 3.4 × 10^−12^ A for 30 wt.%, which is consistent with the trend of polarization current.

## 4. Discussion

**Figure 10:**
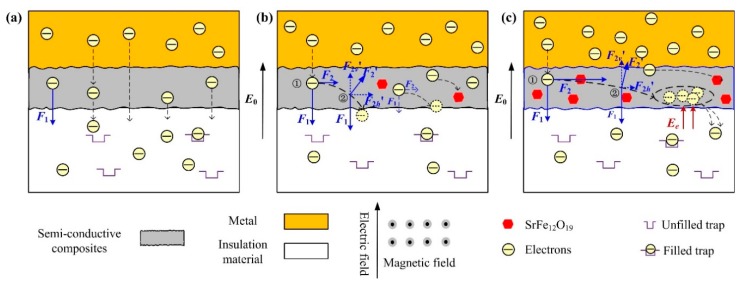
Schematic diagram of electron moving path in the semi-conductive layer under the combined action of electric field and magnetic field. (**a**) Without SrFe_12_O_19_ in the semi-conductive layer; (**b**) low SrFe_12_O_19_ content in the semi-conductive layer; and (**c**) high SrFe_12_O_19_ content in the semi-conductive layer.

The experimental results (space charge distribution, polarization current, and depolarization current) indicate that the semi-conductive composites containing a small amount of SrFe_12_O_19_ will inhibit the charge injection from the metal electrode into the insulation sample to some extent. Because the moving charges in the semi-conductive layer are affected by electric field and magnetic field, the result is the deflection of the charge motion path. Figure 10 shows a schematic diagram of charge movement path in the SrFe_12_O_19_/semi-conductive composites under the combined action of electric field and magnetic field. 

Under the strong electric field, the free charges in the metal electrode will hop through the interface barrier and migrate towards the insulation sample. The moving charges are affected by the electric field force F_1_, and most of them will be injected into the insulation sample along the direction of electric field, and their movement trajectory is approximately straight, as shown in Figure 10a.

When a small amount of SrFe_12_O_19_ is doped into the semi-conductive layer, the experimental results show that the charges in the insulation sample are suppressed to some extent. When the content is 5 wt.%, the insulator sample has the smallest charge amount, 0.89 × 10^−7^ C, decreasing by about 37%, and the steady-state current is 6.01 × 10^−10^ A, decreasing by about 22%. Because the moving charges are deflected by Lorentz force when they pass through the semi-conductive composites. The schematic diagram of electron movement is shown in Figure 10b. When movement charges pass through the semi-conductive layer doped with the SrFe_12_O_19_, they are affected by both the electric field force F_1_ and the magnetic field force F_2_. Assume that the electrons in the metal are incident perpendicular to the insulation sample, and the initial rate is V0. When the movement electrons pass through the SrFe_12_O_19_/semi-conductive composites, they are subjected to the horizontal Lorentz force F_2h_’ and the vertical downward electric field force F_1_ at the initial position ①. The direction of electron movement will be deflected under the resultant forces, arriving at the position ②. At the position, the direction of Lorentz force F_2_ changes, correspondingly, due to the change of electron movement direction, and the direction and magnitude of the applied electric field F1 remain unchanged. The component of the Lorentz force F_2_′ along the vertical direction F_2v_’ will offset part of the vertical downward electric field force F_1_, resulting in the electrons moving towards the insulation sample being blocked and some electrons unable to enter the insulation sample. 

The movement electrons suffer resistance along the vertical direction and deflect along the horizontal direction, which is related to the magnetic particle content. The experimental results show that when SrFe_12_O_19_ content is high, the effect of the SrFe_12_O_19_/semi-conductive composites on inhibiting electron injection is not obvious and even leads to the increase of electron in the insulation sample. The polarization current and depolarization current in the insulation sample for SrFe_12_O_19_ content of 30 wt.% are significantly higher than those without doping SrFe_12_O_19_. Because the addition of many magnetic particles will produce a larger magnetic induction intensity, and then generate a larger Lorentz force, leading to a large deflection along the horizontal direction. Most movement electrons cannot enter into the insulation sample and will accumulate in the SrFe_12_O_19_/semi-conductive composites, resulting in electric field distortion and the secondary injection of electrons, as shown in Figure 10c. Most of movement electrons are subjected to great resistance and cannot move directly downward but experience a long deflection along the horizontal direction. A large number of electrons will stay in the SrFe_12_O_19_/semi-conductive composites, resulting in space charge accumulation, because the SrFe_12_O_19_/semi-conductive composites is not grounded. Although these electrons cannot be injected directly into the insulation sample, the large number of electrons accumulation will cause local electric field distortions. Especially for the interface between the semi-conductive layer and the insulation sample, the electric field created by the accumulated charge E_e_ has the same direction with the applied electric field E_0_. The interface electric field will be enhanced and result in the secondary injection of electrons.

## 5. Conclusions

In the work, the research idea of changing the injection path of moving charges within a semi-conductive layer by magnetic particles was proposed. The influence of semi-conductive matrix modified by SrFe_12_O_19_ on charge injection characteristics in the insulation sample were studied. The experimental results show that a small amount of SrFe_12_O_19_ can significantly reduce charge injection in the insulation sample. The conclusions are drawn as follows:

(1) When SrFe_12_O_19_ content is low (1 wt.% and 5 wt.%), the experimental results show that the charges in the insulation sample are significantly inhibited. For SrFe_12_O_19_ content with 5 wt.%, the insulation sample has the smallest charge amount, 0.89 × 10^−7^ C, decreasing by 37%, and the steady-state current is 6.01 × 10^−10^ A, decreasing by 22%. This is because the moving charges will be affected by the horizontal Lorentz force, leading to the deflection of the charge migration path in SrFe_12_O_19_/semi-conductive composites. In addition, the moving charges are subjected to the electric field force in the vertical direction, and only some of the electrons enter the insulation sample.
(2) When SrFe_12_O_19_ content exceeds 10 wt.%, the charge suppression effect is not obvious and even leads to the increase of charge amount in the insulation sample, owing to the secondary injection of charges. For high doping content, a large Lorentz force will be generated in the SrFe_12_O_19_/semi-conductive composites, and most moving charges will deflect towards the horizontal direction and cannot directly access the insulation sample, resulting in a large number of charges accumulating in the SrFe_12_O_19_/semi-conductive composites. Consequently, the interface electric field between the semi-conductive layer and the insulation sample was enhanced, leading to the secondary injection of charges.
